# Anaerobic carboxydotrophic bacteria in geothermal springs identified using stable isotope probing

**DOI:** 10.3389/fmicb.2015.00897

**Published:** 2015-09-01

**Authors:** Allyson L. Brady, Christine E. Sharp, Stephen E. Grasby, Peter F. Dunfield

**Affiliations:** ^1^Department of Biological Sciences, University of CalgaryCalgary, AB, Canada; ^2^Geological Survey of CanadaCalgary, AB, Canada

**Keywords:** carboxydotrophs, stable isotope probing, geothermal, carbon monoxide (CO), thermophile

## Abstract

Carbon monoxide (CO) is a potential energy and carbon source for thermophilic bacteria in geothermal environments. Geothermal sites ranging in temperature from 45 to 65°C were investigated for the presence and activity of anaerobic CO-oxidizing bacteria. Anaerobic CO oxidation potentials were measured at up to 48.9 μmoles CO g^−1^ (wet weight) day^−1^ within five selected sites. Active anaerobic carboxydotrophic bacteria were identified using ^13^CO DNA stable isotope probing (SIP) combined with pyrosequencing of 16S rRNA genes amplified from labeled DNA. Bacterial communities identified in heavy DNA fractions were predominated by *Firmicutes*, which comprised up to 95% of all sequences in ^13^CO incubations. The predominant bacteria that assimilated ^13^C derived from CO were closely related (>98% 16S rRNA gene sequence identity) to genera of known carboxydotrophs including *Thermincola, Desulfotomaculum, Thermolithobacter*, and *Carboxydocella*, although a few species with lower similarity to known bacteria were also found that may represent previously unconfirmed CO-oxidizers. While the distribution was variable, many of the same OTUs were identified across sample sites from different temperature regimes. These results show that bacteria capable of using CO as a carbon source are common in geothermal springs, and that thermophilic carboxydotrophs are probably already quite well known from cultivation studies.

## Introduction

Carbon monoxide (CO) is an odorless gas toxic to many animals due to its competitive binding to hemoglobin (Haab, 1990). It has been estimated that about 3.3 × 10^9^ metric tons of CO are released annually to the atmosphere (Conrad, 1996). There are numerous natural biogenic and abiogenic sources of CO. Thermal decomposition and photochemical degradation of organic compounds are important sources of abiotic CO (Sipma et al., 2006). CO is also a component of volcanic emissions, which may contain as much as 1–2% of CO per volume of total gas (Giggenbach, 1980; Svetlichny et al., 1991a; Sokolova et al., 2009 and references therein). Biogenic CO may also be produced in microbial ecosystems, and net CO production has been reported for marine algae (Conrad, 1988) and hypersaline cyanobacterial mats (Hoehler et al., 2001) during photosynthesis. Sulfate-reducing bacteria (SRB) have also been shown to produce CO during fermentation (Voordouw, 2002). Some microbes growing in high temperature environments are likely capable of growth at low concentrations of CO (Sokolova et al., 2009). It has also been suggested that CO-oxidizing microbes may occupy micro-niches in which biogenic CO locally accumulates to high levels (Techtmann et al., 2009).

Microorganisms that have the ability to oxidize CO are termed “carboxydotrophs” (King and Weber, 2007). A number of aerobic and anaerobic bacteria as well as some anaerobic archaea (e.g., methanogens) are capable of using CO as a source of energy and/or carbon (e.g., Mörsdorf et al., 1992; Oelgeschläger and Rother, 2008; Sokolova et al., 2009). Carboxydotrophic energy generation employs the enzyme CO dehydrogenase (CODH) that oxidizes CO to CO_2_, generating electrons. The aerobic and anaerobic versions of this enzyme differ. Anaerobic CODH in bacteria is encoded by *cooS* genes (Techtmann et al., 2011) and contains nickel in the active site, while aerobic CODH is encoded by *cox* genes and contains molybdenum (e.g., Dobbek et al., 2001; King and Weber, 2007). Within the Domain *Bacteria*, anaerobic carboxydotrophs are typically found within the phylum *Firmicutes* and some in the class *Alphaproteobacteria* of the *Proteobacteria* (see Techtmann et al., 2009). Purple non-sulfur bacteria (i.e., phototrophic *Alphaproteobacteria*) exist among the known anaerobic CO oxidizers and were among the first discovered (Uffen, 1981; Kerby et al., 1995). However, an increasing number have been identified that are strictly anaerobic thermophiles belonging to the phylum *Firmicutes* (e.g., Svetlichny et al., 1991b; Sokolova et al., 2002). Hydrothermal systems have been proposed as early ecosystems supporting chemolithotrophic life, including thermophilic anaerobic bacteria and archaea using CO as an energy and carbon source (e.g., Cavicchioli, 2002; Wächtershäuser, 2006; King and Weber, 2007). Examples of thermophilic archaea that use CO include *Thermococcus* sp. NA1, capable of both heterotrophic and carboxydotrophic growth (Lee et al., 2008) and *Archaeoglobus fulgidus* capable of using CO as an autotrophic growth substrate (Henstra et al., 2007a).

While the anaerobic oxidation of CO may be coupled to a variety of respiratory processes such as sulfate reduction and acetogenesis (Oelgeschläger and Rother, 2008), hydrogenogenic carboxydotrophs make up the majority of thermophilic CO oxidizing microbes that have been identified in geothermal environments (e.g., Svetlichny et al., 1991a,b; Sokolova et al., 2004, 2005; Slepova et al., 2006). These bacteria oxidize CO via the water-gas-shift reaction (Uffen, 1981; Sipma et al., 2006):

(1) $$\begin{array}{r} \text{CO}+{\text{H}}_{2}O\to CO_{2}+H_{2}\;\left(\Delta G^{0}=-20kJ\right) \end{array}$$

Thermophilic bacteria and archaea with the capacity for hydrogenogenic carboxydotrophy have been isolated from various locations around the world including the Kunashir Island, Russia (Svetlichny et al., 1991a), Kamchatka (Sokolova et al., 2002; Slepova et al., 2006), Yellowstone National Park (Sokolova et al., 2004), and Iceland (Novikov et al., 2011). The isolates share similar ranges of optimal pH (ca. 6.8–7.0) and temperature (ca. 55–83°C) (see Henstra et al., 2007b; Techtmann et al., 2009) but are phylogenetically divergent (Techtmann et al., 2009). Unlike mesophilic carboxydotrophs, the thermophilic hydrogenogenic species isolated so far do not show growth inhibition by high levels of CO. In fact, most grow under atmospheres of 100% CO, far above natural CO concentrations in geothermal systems (e.g., Svetlichny et al., 1991a,b).

CO oxidizing thermophiles are of potential biotechnological interest for the anaerobic fermentation of synthesis gas (“syngas”). Syngas is a product comprised mostly of H_2_, CO, and CO_2_ resulting from the high temperature gasification of waste biomass, into higher-value bioalcohol fuel (Henstra et al., 2007b). As syngas is produced at high temperatures, bacteria from geothermal sites are of particular interest due to the expected high rates of substrate conversion at high temperatures. Characterization of anaerobic CO-oxidizing bacteria in geothermal systems therefore could provide fundamental information about the natural diversity of thermophilic carboxydotrophs available for these biotechnological applications. DNA-Stable Isotope Probing (SIP) is a valuable tool in assessment of functional bacterial groups. It has been used in a variety of environments to identify active consumers of substrates such as methane (He et al., 2012; Sharp et al., 2012, 2014b). There are however some difficulties in applying SIP to identify carboxydotrophs. Firstly, while some carboxydotrophic bacteria directly incorporate CO-carbon into the carboxyl group of acetate via acetyl-CoA synthases (ACSs) using the Wood-Ljungdahl pathway (e.g., Henstra et al., 2007b), others incorporate the CO_2_ produced from CO oxidation as the direct source of cellular carbon, using reductive CO_2_ pathways such as the Calvin-Benson-Bassham (CBB) Cycle, or the reverse tricarboxylic acid (TCA) cycle (Uffen, 1981; Ragsdale, 1991, 2004; Berg, 2011). As such, CO_2_ present in the atmosphere may be incorporated rather than the CO_2_ produced directly from the oxidation of CO diluting the labeling effect. Another issue with CO-SIP is that the products of CO oxidation, i.e., H_2_ and CO_2_, may result in labeling of other autotrophs. Cross-feeding is a caveat in any SIP experiment, but the severity of the cross-feeding, especially via CO_2_, can often be assessed with controls such as the addition of exogenous ^12^CO_2_ (e.g., Sharp et al., 2012). CO-SIP will also not detect carboxydoheterotrophs that may oxidize CO but use a different carbon source. Therefore, in this study we assessed the value of CO-SIP to identify anaerobic thermophilic carboxydoautotrophic bacteria in some geothermal springs.

## Materials and methods

### Geothermal spring sample collection

Five geothermal springs were selected for CO-SIP investigations from among geothermal sites in Western Canada (Grasby et al., 2000; Sharp et al., 2012). Soil or sediment or biomat samples were collected at various times of the year between fall 2010 and fall 2012 into sterile screw-cap tubes (Table 1). Samples were kept cold as soon as possible to minimize changes in the microbial community during transport. Collected material was sub-sampled within approximately 5 days of sampling for DNA extraction, and the remainder was stored at 4°C for 1–2 d prior to incubation studies.

**Table 1:** **Name of geothermal spring, measured *in situ* pH and temperature, and anaerobic CO-oxidation potentials for sites included in the current study**.

**Geothermal site**	**Sample ID**	**Description**	**Environmental temperature (°C)**	**Incubation temperature (°C)**	**pH**	**CO oxidation potentials (μmol CO g^−1^ d^−1^)**
Dewar Creek	DCm2010	biomat	54.9	55	8.30	20.4 ± 1.8
	DCmN11	biomat	45.0	45	8.30	24.6 ± 1.1
	DCs9	sediment	64.7	65	7.94	48.9 ± 5.4
Lakelse	L3	organic rich sediment	45.1	55	8.27	13.6 ± 2.7
Grayling River	GR1	sediment	56.1	55	7.02	18.6 ± 4.2
Liard	Liard2	biomat	53.5	55	6.76	30.2 ± 1.0
Portage Brûlé	PB1	biomat	45.9	45	6.34	35.7 ± 3.7

*Oxidation potentials represent duplicate incubations and are listed in μmol CO g^−1^ (wet weight) d^−1^ with one s.d*.

### Soil microcosms and CO oxidation

Approximately 2–5 g of sample material (wet weight) was added to 120-ml serum bottles and crimp-sealed with sterile blue butyl rubber stoppers. An anoxic environment was created by repeated (3 ×) evacuation and refilling with N_2_ gas. CO was added to final mixing ratios of 5–10% in the headspace to assess oxidation potential and act as a ^12^C incubation for stable isotope probing (SIP) experiments. For SIP experiments, labeled gases ^13^CO (99 atom % ^13^C, Sigma Aldrich) and ^13^CO_2_ (99 atom % ^13^C, Sigma Aldrich) were used at mixing ratios of 10% v/v, both separately and in combination with non-labeled CO and CO_2_ gases in different SIP trials. “Control” is used to refer to the un-incubated (i.e., no CO or CO_2_ added) environmental samples. Microcosms were incubated at close to environmental temperatures (Table 1). Headspace CO was monitored at ca. 1-d intervals using a Varian 450-Gas Chromatograph equipped with a 0.5-m Hayseep N and a 1.2-m Mol Sieve 16X column in series coupled to a Thermal Conductivity Detector (GC/TCD). Potential production of methane was monitored using a GC-Flame Ionization Detector (FID). Incubations proceeded until approximately 95–100% of the added CO had been consumed, typically within a week. Samples were then harvested and frozen immediately at −85°C. CO consumption rates were based on duplicates of any sample (^12^CO and ^13^CO only incubations), while duplicate SIP experiments were performed only in some cases.

### DNA extraction and density fractionation

DNA was extracted from approximately 500 mg of sample using the FastDNA Spin Kit (MP Biomedicals) with the addition of washing steps using guanidine thiocyanate (Knief et al., 2003). Quantification was performed using the Quant-iT™ dsDNA HS Assay Kit (Invitrogen) and extracted DNA was stored at −20°C prior to ultracentrifugation separation. Heavy and light DNA were separated by density gradient ultracentrifugation using cesium chloride (CsCl) as described by Neufeld et al. (2007), with minor modifications. Five hundred nanograms to one microgram of total DNA was typically used for each SIP assay. To account for any variability in DNA distribution patterns that may arise due to using inconsistent amounts of DNA in CsCl gradients, within an individual sample set (i.e., all trials from the same sample site) similar total DNA amounts were used. Centrifugation and gradient fractionation were performed as described by Sharp et al. (2014b). DNA was precipitated from each fraction using polyethylene glycol (PEG) and glycogen as in Neufeld et al. (2007). DNA present in each density gradient was quantified using the Quant-iT™ dsDNA HS Assay Kit (Invitrogen). As the focus of this paper is assessing bacterial rather than archaeal carboxydotrophy, 16S rRNA gene PCR assays for each fraction were set-up using a QIAgility (v. 4.13.5) with bacterial specific primers 519f and 907r (Stubner, 2002). 16S rRNA gene copies were quantified on a Rotor-Gene Q (Qiagen) as in Sharp et al. (2012).

### Microbial community analysis

The density gradients of DNA extracted from incubations with ^13^C-labeled vs. ^12^C-labeled substrates were compared. Heavy SIP fractions with increases in the relative amounts of DNA and/or 16S rRNA gene copies were selected for pyrosequencing analysis of the 16S rRNA gene. “Light” fractions (density ca. 1.690 g ml^−1^) from ^13^CO incubated samples were also analyzed in some cases for comparison to heavy fractions (Table S1). Where possible, DNA was also amplified from the corresponding heavy fractions from untreated controls (**Figure 2**, Table S1). However, in some cases the amount of DNA present in the fractions was below detection, or too low to obtain enough for pyrosequencing (e.g., DCm2010) (Supplementary Figure S1). Samples were prepared for sequencing analysis as described previously (Grasby et al., 2013; Sharp et al., 2014a) using FLX Titanium amplicon primers 454T_RA_X and 454T_F, which contain 16S rRNA gene targeted primers 926fw (5′-aaactYaaaKgaattgRcgg-3′) and 1392r (5′-acgggcggtgtgtRc-3′) designed to target both bacteria and archaea (Ramos-Padrón et al., 2011). PCR reactions and purification were performed as described in Sharp et al. (2014a). Purified PCR products (ca. 150 ng total DNA) were analyzed at the Genome Quebec and McGill University Innovation Centre, Montreal, Quebec on a 454 Life Sciences Genome Sequencer FLX (Roche) machine running the Titanium chemistry.

### Sequence data processing

Quantitative Insights Into Microbial Ecology (QIIME) pipeline version 1.8 (Caporaso et al., 2010) was used to process raw sequence data as in Sharp et al. (2014a). A minimum quality score of 25 was used and sequences were screened using ChimeraSlayer (Haas et al., 2011). Taxonomic identification of a representative sequence (most common) for each phylotype (clustered at 97% similarity) was determined using nucleotide Basic Local Alignment Search Tool (BLAST) (Altschul et al., 1990) against the Silva 111 reference database (Pruesse et al., 2007). Eukaryotic and chloroplast sequences were removed from further analysis. Final numbers ranged from 2195 to 21118 sequences per sample (Table S1). A phylogenetic tree was constructed using the parsimony-add function in ARB (Ludwig et al., 2004).

16S rRNA gene sequences obtained from this study have been deposited in the SRA database under accession numbers SRP028305 and SRP059036. Representative sequences of identified OTUs present at 25 fold enrichment are provided in the Supplementary Material.

## Results

### Biodegradation of carbon monoxide

Anaerobic oxidation of carbon monoxide was detected via monitoring of the CO mixing ratio in serum bottle headspaces after adding CO. Communities that showed evidence for CO consumption were selected for further investigation using stable isotope probing (SIP). The average values for anaerobic CO oxidation potentials for each sample is listed in Table 1. Rates ranged from 13.6 to 48.9 μmol CO g^−1^ d^−1^. The fastest rates were observed in samples from the Dewar Creek hot spring. Despite the anaerobic conditions, no methane production was observed in sample sites GraylingRiver1, Liard2, and PortageBrûlé1 during any incubation and only minor amounts were observed in other Dewar Creek and Lakelse samples, with estimated CH_4_ production rates of 0.013–11.4 nmol mol day^−1^ g^−1^. The amount of CH_4_ produced corresponds to ca. < 0.1% of the added CO being converted to methane. One exception was a Lakelse sample that produced more total CH_4_ (~ 18 μmol) than CO added, indicating other substrates were present for methanogenesis.

### Identification of active CO consuming bacteria using SIP combined with 16S rRNA gene sequencing

**Figure 1:**
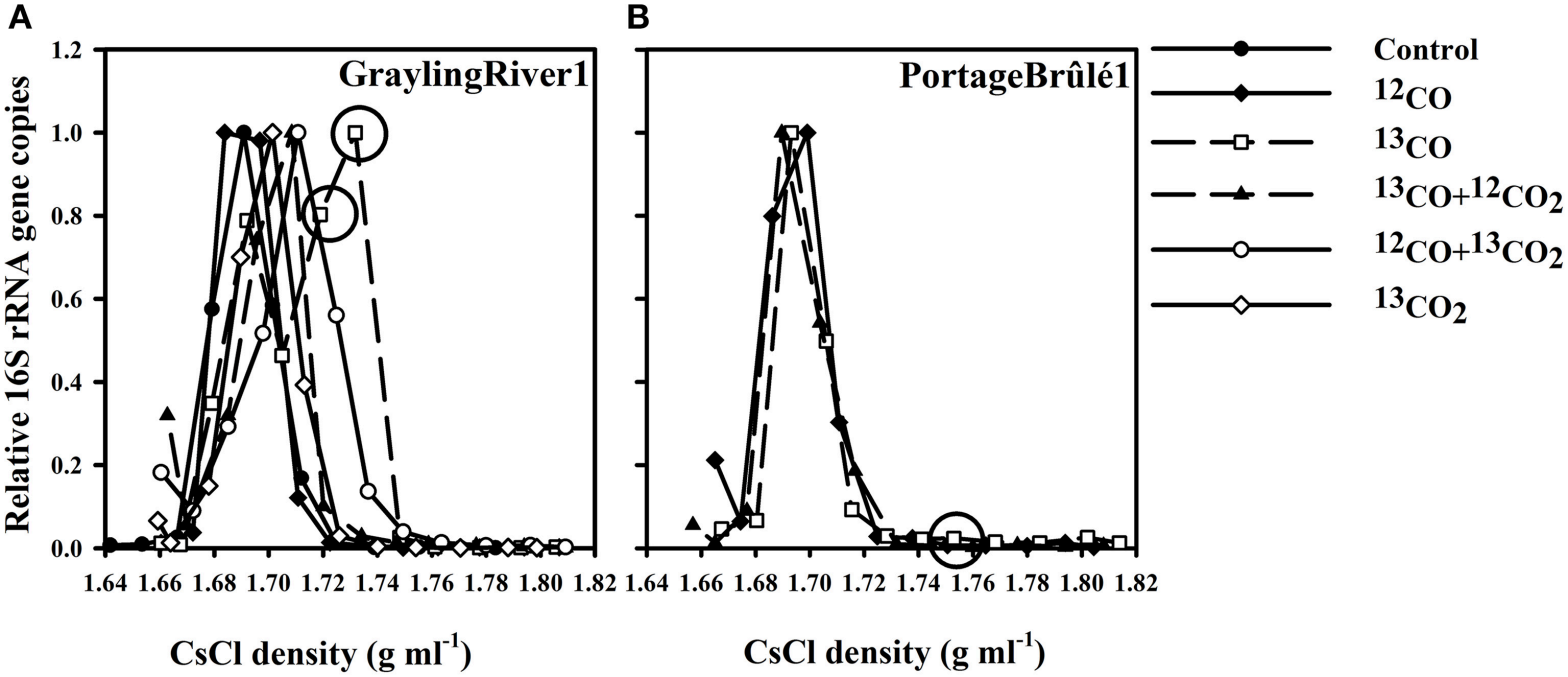
**(A)** Representative 16S rRNA gene counts vs. DNA density for SIP experiments using GraylingRiver1 sediment, illustrating the shifts in relative 16S rRNA gene copies associated with different incubations. The incubations using only ^13^CO as the sole carbon and energy source showed the greatest increase in the relative number of 16S rRNA gene copies in heavy fractions at densities of approximately 1.730 g ml^−1^. DNA from these heavy fractions was used for 16S rRNA gene pyrosequencing. **(B)** Relative 16S rRNA gene copies from PB1 sediment showing no density shift with ^13^CO incubations despite active CO oxidation. Circles indicate ^13^CO incubation fractions used for 16S rRNA gene pyrosequencing analyses. “Control” represents the un-incubated (no CO or CO_2_ added) environmental sample.

Density profiles of DNA extracted from samples after incubation with ^13^CO generally ranged from 1.660 to 1.800 g ml^−1^. Shifts in DNA density compared to un-amended control samples were often subtle (Supplementary Figure S1). Therefore, quantitative real-time PCR (qPCR) counts of total bacterial 16S rRNA gene abundance were used to identify density fractions that had an increase in the relative number of gene copies (Figure 1, Supplementary Figure S2).

Representative profiles of 16S rRNA gene copies vs. DNA density for GraylingRiver1 (GR1) and PortageBrûlé1 (PB1) SIP experiments are shown in Figure 1. The number of 16S rRNA gene copies in the control environmental communities peaked in the density range of 1.690–1.700 g ml^−1^. Incubations using ^12^CO showed the same peaks, demonstrating that incubation with CO itself had little effect on the overall DNA density profile of the community. However, incubation of GR1 with ^13^CO resulted in a marked shift in 16S rRNA gene copies toward heavier fractions, indicating assimilation of the ^13^C from ^13^CO into DNA. In contrast, no significant shift in DNA density or 16S rRNA gene copies was observed over multiple incubations of the PB1 sample under ^13^CO. Although this sample also oxidized CO, no assimilation of ^13^C was evident. These two samples were representative of the two major patterns observed. Other samples analyzed are shown in Supplementary Figures S1, S2.

A fundamental issue with interpreting SIP results is cross-labeling of other bacteria via metabolic products, especially CO_2_. The severity of this problem can be estimated via several controls. In GR1 (Figure 1A), as well as in several other samples tested (Supplementary Figures S1, S2) incubations with only ^13^CO_2_ and no added CO always showed very minor density shifts in DNA and 16S rRNA gene counts. This indicated that other autotrophs growing on substrates such as sulfur and ammonia were of minor importance. CO was therefore the primary energy source in the incubations, and the food webs detected were ultimately based on CO oxidation.

In samples like GR1, microcosms containing ^13^CO as the sole carbon and energy source (with no CO_2_ addition) displayed the greatest shift in density (Figure 1). Less of a shift in density was observed in incubation with ^13^CO + ^12^CO_2_, probably because assimilation of ^12^C from the added CO_2_ diluted the labeling effect. Incubation with ^12^CO + ^13^CO_2_ showed some increase in 16S rRNA gene copies compared to the un-incubated control, albeit to a lesser extent than in ^13^CO. The apparent assimilation of C preferably from ^13^CO but also from ^13^CO_2_ indicates that CO_2_ was probably the primary C source of the CO oxidizers, but that there may be a diffusion effect whereby the ^13^CO_2_ produced directly by a carboxydotroph from ^13^CO is preferentially assimilated compared to exogenous ^12^CO_2_ supplied in the atmosphere.

In the majority of cases, the “heavy” fractions that showed the greatest increase in 16S rRNA gene copies were at a density of ca. 1.730 g ml^−1^ (Figure 1, Supplementary Figure S2). Heavy fractions that contained a large increase in DNA amounts and/or 16S rRNA gene copies as compared to an un-incubated, non-labeled control were selected for 16S rRNA gene sequencing. In some cases (e.g., DCm2010), the amount of DNA present in the corresponding high-density fractions of the unlabeled controls was too low to obtain enough for successful pyrotag sequencing despite inputs of similar total amounts of DNA (see Supplementary Figure S1).

**Figure 2:**
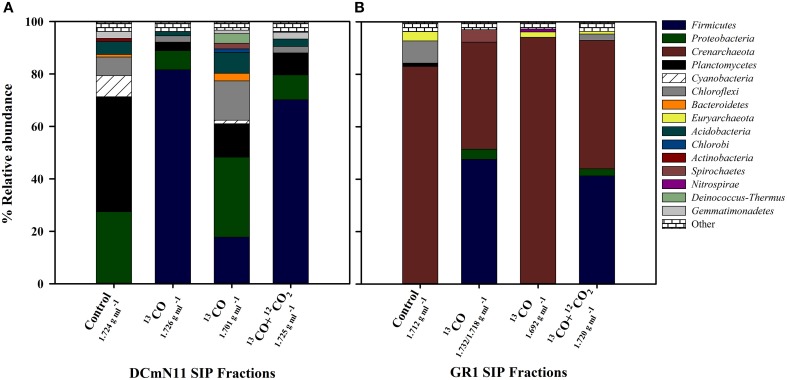
**Relative abundance (%) of different phyla for SIP incubations showing results from heavy (density ca. 1.730 g ml^−1^) fractions and light fractions (density ca. 1.700 g ml^−1^) of samples incubated with ^13^CO or ^13^CO + ^12^CO_2_ compared to the community detected in heavy fractions of un-incubated Control samples (i.e., no CO or CO_2_ added)**. **(A)** DCmN11 showing significant increase in the proportion of *Firmicutes* in heavy fractions and **(B)** GR1 showing an increase in *Firmicutes* but also the large proportion of *Crenarchaeota* present in both the un-incubated environmental control sample and SIP fractions. 16S rRNA gene sequences were clustered at 97% similarity and classified using QIIME. “Other” includes phyla present at < 1%.

Most predominant operational taxonomic units (OTUs) in the heavy fractions of all samples (i.e., most putative carboxydotrophs) belonged to the phylum *Firmicutes*, in particular to the class *Clostridia*. In heavy fractions recovered from ^13^CO incubations that showed observable shifts in 16S rRNA gene copies, members of the phylum *Firmicutes* accounted for 31–95% of the reads (Table S1). One of the lowest % of *Firmicutes* was from GR1 which had a very high proportion of *Crenarchaeota* in both the original community and in all heavy fractions. However, of bacterial sequences only, *Firmicutes* accounted for 81.5% in the heavy fraction of GR1_^13^CO. The proportions of top phyla (>1% of sequences) of both the un-amended environmental samples and the heavy fractions for two sites that showed strong DNA labeling are shown in Figure 2.

**Figure 3:**
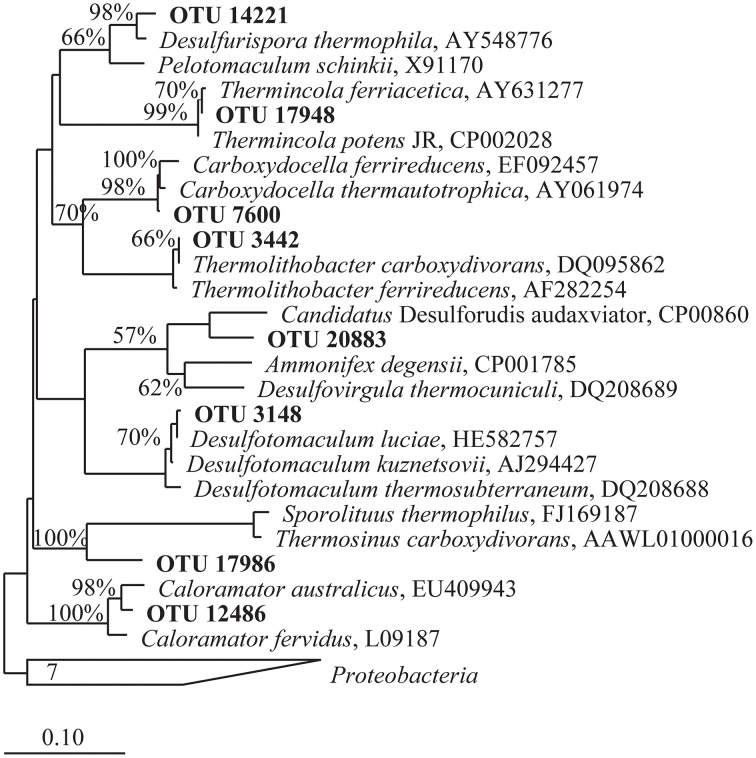
**Phylogenetic tree of partial 16S rRNA gene sequences belonging to OTUs identified in heavy fractions from ^13^CO SIP incubations present at a minimum of 25 fold enrichment**. OTUs presented are from those incubations that showed an observable shift in density and are hypothesized to represent microbes that oxidized CO. A skeleton tree was constructed from near complete 16S rRNA gene sequences (>1400 bp) via Neighbor-joining with a Jukes-Cantor correction and 10,000 bootstraps. Shorter sequences produced via 454 pyrosequencing obtained in this study were added by parsimony using ARB (in bold). The scale bar represents 0.1 change per nucleotide position. Bootstrap support values greater than 55% for the major nodes are given. The tree was rooted using 7 *Proteobacteria* 16S rRNA gene sequences.

The taxonomic identifications of OTUs recovered from^13^CO microcosm heavy fractions that were present at 25 fold enrichment compared to the original environmental sample are shown in Table 2. A 16S rRNA gene phylogenetic tree was constructed using the top OTUs from each ^13^CO heavy fraction showing an observable shift compared to reference sequences (Figure 3). Taxonomic identifications of OTUs present at >1% of all sequences are presented in Supplementary Table S2. Most of the putative carboxydotrophs identified belonged with >98% sequence identity to genera that include known CO-oxidizers, such as *Thermincola, Desulfotomaculum, Carboxydocella*, and *Thermolithobacter* (Sokolova et al., 2002, 2005, 2007; Parshina et al., 2005a,b). For example, the most abundant OTU (OTU_17948) recovered from ^13^CO heavy fractions from Dewar Creek (DCm2010 and DCmN11) and Lakelse springs showed 99% sequence identity to both *Thermincola potens* and to *Thermincola carboxydiphila*, known CO-oxidizing bacteria (Sokolova et al., 2005; Byrne-Bailey et al., 2010) (Table 2). Members of the genus *Carboxydocella* were also detected in the majority of heavy fractions across all sites. This genus was represented by multiple OTUs, however the top OTU_7600 identified in most samples corresponded to *Carboxydocella thermautotrophica* (98% similarity). Members of the genus *Desulfotomaculum* were most predominant in DCs9, a sediment sample collected from Dewar Creek. In DCs9_^13^CO incubations, 31.9% of sequences were attributed to the genus *Desulfotomaculum*. 30.0% of sequences were in OTU_3148, which showed 99% sequence identity to *D. kuznetsovii* and *D. luciae*.

**Table 2:** **The percent of total sequences associated with top OTUs identified in heavy fractions of ^13^CO incubations**.

**OTU**	**Phylum**	**BLAST identification**	**Identity (%)**	**DCm2010 1.733 g ml^−1^**	**DCmN11 1.726 g ml^−1^**	**DCs9 1.740 g ml^−1^**	**L3 1.721 g ml^−1^**	**GR1 1.726 g ml^−1^**	**Liard2 1.734 g ml^−1^**	**PB1 0.753 g ml^−1^**
3148	*Firmicutes*	*Desulfotomaculum kuznetsovii/D. luciae*	99			30.0 (n.d.)				
3442	*Firmicutes*	*Thermolithobacter carboxydivorans*	100			8.9 (n.d.)				
7600	*Firmicutes*	*Carboxydocella thermautrotrophica*	98	7.6 (n.d.)	2.9 (n.d.)	17.9 (0.0)		17.0 (n.d.)	28.0 (n.d.)	
9076	*Firmicutes*	*Sporomusa sphaeroides*	94							15.7 (n.d.)
12486	*Firmicutes*	*Caloramator australicus*	99				7.3 (n.d.)			
14221	*Firmicutes*	*Desulfurispora thermophila*	97		1.6 (n.d.)		7.5 (0.0)			
17597	*Firmicutes*	*Streptococcus thermophilus*	100							26.7 (n.d.)
17948	*Firmicutes*	*Thermincola potens/ T. carboxydiphila*	99	68.2 (n.d.)	65.5 (n.d.)		48.4 (n.d.)	3.9 (n.d.)		
17986	*Firmicutes*	*Thermincola potens/ T. carboxydiphila*	90			8.1 (n.d.)				
18478	*Deinococcus-Thermus*	*Thermus scotoductus*	100			5.4 (0.2)				
20883	*Firmicutes*	*Candidatus* Desulforudis audaxviator	93					15.1 (n.d.)		
21098	*Proteobacteria*	*Azonexus caeni*	98							19.3 (0.2)

*The density of the heavy fraction analyzed is reported in g ml^−1^. OTUs reported are those with a 25 fold enrichment compared to the original community in at least one sample site. The percent of total sequences for each OTU is reported with BLAST identification and percent sequence identity to the top cultured BLAST hit. Numbers in brackets represent the proportion (%) of sequences present in the same OTU in the original environmental sample. n.d., not detected*.

Some OTUs identified may reflect bacteria with as yet unknown or unconfirmed CO-oxidizing capabilities. One such cluster, OTU_20883, present in both the GR1_^13^CO and GR1_^13^CO + ^12^CO_2_ heavy fraction at 15.1 and 5.3% of sequences respectively, is 93% similar to *Candidatus* “Desulforudis audaxviator.” Neither this OTU nor any others that showed any similarity to this bacterium were detected in the control environmental sample from this site. BLAST results for OTU_17986 representing 8.1% of the total for DCs9_^13^CO returned equal results for *Thermincola potens* and *Thermincola carboxydiphila*. However, the sequence identity to both was only 90%, and it branches distantly from *Thermincola* in the phylogenetic tree (Figure 3). This may represent another related CO-oxidizing genus, the exact nature of which requires further study.

While PB1 and Liard2 sediments did oxidize CO, incubations with ^13^CO did not show any observable shift in 16S rRNA gene density profiles. Nevertheless, we did for comparison analyze the heavy fractions from these incubations. The heavy fraction of PB1 showed a slight increase in *Firmicutes* as compared to the proportion present in the original community (Table S1), however no OTUs with sequences >1% were associated with known CO-oxidizing bacteria (Table S2). In comparison, the heavy fraction from Liard2_^13^CO had a number of OTUs associated with CO-oxidizing bacteria. Only one OTU was present at 25 fold enrichment and represented the top OTU of this fraction (Table 2). OTU_7600 had a 98% BLAST identity to *Carboxydocella thermoautotrophica*. It represented 28.0% of sequences and was the same *Carboxydocella* OTU found in other sites at relatively high abundance. Therefore, growth of carboxydotrophs was probably occurring in these samples as well, albeit at slow rates.

High G + C content may be responsible for the observation of some organisms in heavy fractions, including uncultured *Crenarchaeota* present in heavy fractions recovered from GR1_^13^CO (Table 2). The G + C content of previously identified CO-oxidizing thermophiles ranges from ca. 40–48% (Svetlichny et al., 1991b; Sokolova et al., 2002, 2004, 2005), corresponding to a density range of 1.698–1.705 g ml^−1^. Increased G + C content results in a higher buoyant density that may initially suggest ^13^C incorporation (Schildkraut et al., 1962). However, the high proportions in un-amended and ^12^C controls of *Crenarchaeota* in the case of GR1 suggest that these microbes were not carboxydotrophs but rather are naturally present at that density due to relatively high G + C content.

## Discussion

In this study, bacteria potentially involved in the anaerobic oxidation of carbon monoxide were identified from hot spring environments using DNA-SIP. Five geographically diverse geological settings were identified in which potential anaerobic CO-oxidation was detectable. These five locations are widely dispersed geographically over an area of approximately 1 million km^2^. The measured CO-oxidation potentials were variable between geothermal springs. Comparative rates from other environments are rarely reported, however CO-oxidation rates of 120 μmol l^−1^ of sediment d^−1^ were estimated in slurries from Uzon Caldera, Kamchatka (Kochetkova et al., 2011), and a ^14^CO tracer was used to estimate a rate of 40.75 nmol CO cm^−3^ sediment d^−1^ for another anaerobic hot spring community in Kamchatka (Slepova et al., 2007). While obtained following different methodology, these *in vitro* rate estimates are 2–4 orders of magnitude lower than those measured in the present study, and show that potential rates of CO oxidation may vary greatly between environments. The observation of little to no methane production in most samples was consistent with the negligible proportions of *Euryarchaeota* detected in the original environmental communities and the lack of archaeal sequences detected in heavy SIP fractions (e.g., Figure 2A).

Distinct differences were noted between the original microbial communities and the communities detected in ^13^C-labeled heavy DNA fractions for each sample. In most cases, *Firmicutes* were present at < 1% in the original communities but increased in abundance in ^13^CO incubated heavy fractions, reaching up to ca. 95% of all 16S rRNA gene reads (Table S1). These results indicate that CO-metabolizing bacteria make up a relatively minor component of the overall population within these geothermal systems but are still present and may become active if CO is provided. Most of the bacteria identified in heavy fractions showed high identities (>98%) to known CO-oxidizing bacteria described from other geothermal springs, particularly *Carboxydocella* and *Thermincola* species (Table 2). This finding suggests not only that the CO-SIP procedure was successful in identifying primary carboxydotrophs without cross-feeding artifacts, but also that the predominant carboxydotrophic bacteria in geothermal environments may in fact already be well described from cultivation studies. This is a rather unusual finding for SIP experiments (e.g., Redmond et al., 2010). Geographically, it also indicates that anaerobic thermophilic carboxydotrophic bacteria are highly cosmopolitan (at least at the species/genus level), since most described isolates have been obtained from Russian geothermal sites. Aerobic carobyxdotrophy is taxonomically diverse (e.g., King and Weber, 2007). And while the presence of CO in geothermal spring emissions may suggest the potential for wide-spread CO metabolism, the current study supports the notion that the capacity for anaerobic carboxydotrophy among thermophiles is more limited.

While the most predominant bacteria identified via SIP were similar to carboxydotrophs isolated from other geothermal springs, there were a few exceptions. Among the predominant OTUs detected in heavy DNA fractions (Table 2), three OTUs showed < 95% 16S rRNA gene sequence identity to any described species. For example, an OTU making up 15% of the heavy DNA fraction in sample GR1 had only a moderate similarity to the proposed genus “Desulforudis.” *Candidatus* “Desulforudis audaxviator” was identified in the fracture water of a South African gold mine. This isolate has components of the Wood-Ljungdahl pathway and may be capable of CO oxidation and assimilation (Chivian et al., 2008). However, the low identity (93%) of our OTU indicates a genus-level divergence to Ca. “D. audaxviator.”

While the use of 16S rRNA gene qPCR greatly improved the detection of shifts in density within the CsCl gradients, in general the observed shifts were subtle. Approximately 0.47 mmol of total ^13^C was added to each of the ^13^CO SIP incubations. However, previous studies show that relatively little of the CO oxidized microbially in geothermal habitats is incorporated into biomass. Using radioisotope tracers to examine a hot spring community from Kamchatka, it was estimated that 85% of the ^14^CO was oxidized to CO_2_ while only 0.5% was used for cell biomass production. The remainder was distributed between dissolved organic matter and minor (0.001%) amounts of methane (Slepova et al., 2007). At 0.5% incorporation, a maximum of ca. 2.35 μmol of ^13^C would have been incorporated into the bacteria identified in the current study and may explain why shifts in the density of labeled DNA were minor compared to SIP experiments using substrates such as ^13^CH_4_ where more C is incorporated into the cells over a short incubation period (Dumont et al., 2011). The use of qPCR and the examination of the 16S rRNA gene copy profiles provided a means by which to identify subtle shifts in DNA density (Lueders et al., 2004; Sharp et al., 2012). The lack of observable shifts in 16S rRNA gene copies in one of our samples (^13^CO-incubated PortageBrûlé) indicated that some geothermal communities may contain bacteria that are using CO as an energy source to maintain the population but are perhaps growing slowly, maybe due to other nutrient limitations, or more likely are incorporating other, possibly organic, C sources into biomass (Figure 1B). This does indicate limitations in the ^13^CO-SIP technique. While it appeared to be effective in identifying some carboxydotrophs, it cannot identify all of these metabolically diverse microorganisms including potential carboxydoheterotrophs.

The ^13^CO-SIP technique is also challenging because carboxydotrophs may incorporate CO_2_ rather than CO directly, and because the initial products of CO-oxidation, H_2_ and CO_2_, may lead to labeling of other autotrophs. Our experiments included controls suggesting that these problems were minor. The greatest shifts in density were observed when ^13^CO was provided as the sole carbon source (i.e., no extra CO_2_ was added), but incubations with ^13^CO + ^12^CO_2_ showed smaller shifts in DNA density compared to ^13^CO alone. Most likely, an initial oxidation of ^13^CO to ^13^CO_2_ via the gas-water shift reaction is followed by assimilation of the produced ^13^CO_2_. Incubations with ^13^CO_2_ alone (and no CO added) showed little or no apparent shifts in DNA density, indicating that labeling of autotrophs growing on substrates already present in the samples, such as sulfur or ammonia, was not an issue, and that CO was ultimately the primary energy source in the incubations. However, there is the distinct possibility of hydrogenotrophic organisms using the H_2_ and ^13^CO_2_ produced via the gas-water shift reaction. This particular form of cross-feeding cannot be eliminated from the current results, but lines of evidence suggest that it was minor: (1) Cross-feeding with H_2_ may occur only in addition to primary CO oxidation- i.e., it can only be a secondary process given that CO was the major energy source available; (2) almost all of the detected bacteria in the present study were closely related to known carboxydotrophs; and (3) potentially hydrogenotrophic but non-carboxydotrophic bacteria such as the members of the phylum *Deinococcus-Thermus* were detected in some heavy DNA fractions, but were always of minor importance compared to known carboxydotrophs (e.g., *Thermus scotoductus* in DCs9, Table 2; Table S2). Complete genomes of both *T. scotoductus* and *T. antranikianii* (Table S2) lack genes related to CO metabolism (http://img.jgi.doe.gov/).

Many bacterial species that possess *cooS* genes have a primary metabolism that does not focus on CO, however their presence may imply a potential underlying or backup CO-dependent physiology should conditions vary and become more optimal for CO-oxidation (Techtmann et al., 2011). While challenging to measure *in situ*, localized accumulations of CO may create microniches within geothermal systems in which low abundance carboxydotrophic population members may thrive. For example, the species composition of the heavy fractions was similar for two biomat samples DCm2010 and DCmN11 with a dominance of *Thermincola potens* in both cases. The presence of CO oxidizing bacteria in these samples is perhaps not surprising given the observation of net CO production within microbial mats. Saline and intertidal sand flat photosynthetic microbial mats exhibited a net production of CO (3.1–5.4 μmol m^−2^ d^−1^) during daylight hours and were also observed to have a net production of H_2_ (Hoehler et al., 2001). As many of the Dewar Creek samples that showed evidence of CO-oxidation were comprised of biomats with relatively high proportions of cyanobacteria (Figure 2B, Table S1), these results support CO as a potentially important carbon and energy source in microenvironments within geothermal systems. Variation in CO metabolizing bacteria was observed between samples collected from the same geothermal system but under different temperature regimes. OTU_3148 was 99% similar to both *Desulfotomaculum kuznetsovii* and *Desulfotomaculum luciae*, detected in ^13^CO incubations of DCs9, another site from the Dewar Creek hot spring. *Desulfotomaculum kuznetsovii* is an obligate anaerobe and is capable of growth with CO as the sole carbon and energy source (Parshina et al., 2005a). *Thermolithobacter carboxydivorans* was also detected in this particular sample site but was not detected in any other microcosm. The optimum growth temperature for *T. carboxydivorans* of 70°C may also explain the presence of this bacterium in DCs9 with an environmental and incubation temperature of 65°C as opposed to other Dewar Creek samples with lower *in situ* temperatures incubated at 55°C (Sokolova et al., 2005).

The dominance of *Firmicutes* in heavy-density DNA fractions of geothermal samples incubated under ^13^CO confirms that representatives of this phylum may play a predominant role relative to other phyla in anaerobic oxidation of CO in geothermal environments. In particular, they may reflect minor populations within geothermal microenvironments where localized CO concentrations may be high. While the detection of such microenvironments *in situ* is challenging, the hypothesized presence of these localized CO-rich niches (e.g., Techtmann et al., 2009) suggests a mechanism by which these carboxydotrophs may exist. Despite comprising a relatively small proportion of *in situ* communities, the CO oxidizing bacteria are active and show some variation across geothermal environments. Oxidation potentials are higher than the few previously reported rates for mixed geothermal communities. Despite geographical differences, thermophilic bacteria associated with anaerobic CO-oxidation are widely distributed geographically and the predominant species are well-described from cultivation studies. The presence of a few OTUs that do not show high degrees of similarity to any known cultured representatives indicates that a few new lithoautotrophs that have not been previously identified as CO-oxidizers may also be present in the geothermal springs tested and require further study.

The detection of sequences associated with known CO-oxidizing bacteria in high abundance supports the applicability of the CO-SIP technique. SIP can be applied to target autotrophs by adding an energy substrate along with ^13^CO_2_. This works as long as the added substrate is the primary energy source for the autotrophic community. We have previously used this approach to identify autotrophic methanotrophs (Sharp et al., 2012). We therefore conclude that the CO-SIP technique, which works in a similar way to identify autotrophic carboxydotrophs, does have some value, although of course the results still need to be interpreted with caution. Controls are necessary to demonstrate a low rate of assimilation of ^13^CO_2_ by other autotrophs. It should also be stressed that heterotrophic carboxydotrophs will be missed- this was a possible explanation for the failure of some of the samples assayed in this study. Cross feeding via H_2_ + CO_2_ produced via the gas-water shift reaction is also a potential issue, although this appeared to be minor in this particular study. This study appeared to work because the bacteria identified were primarily known carboxydotrophs, however identification of a new potential carboxydotroph should only be taken as initial evidence that requires verification with other methods including sequencing of potential *cooS* genes.

## Author contributions

AB, CS and SG collected samples; AB performed SIP incubations with input from PD; AB with aid from CS extracted DNA, prepared samples for sequencing and carried out data processing. AB and PD wrote the initial draft of the paper; all authors designed the study, discussed the results and commented on the manuscript.

### Conflict of interest statement

The authors declare that the research was conducted in the absence of any commercial or financial relationships that could be construed as a potential conflict of interest.
