# Supplementary figures and images for: Anaerobic carboxydotrophic bacteria in geothermal springs identified using stable isotope probing
Source: Front Microbiol. 2015 Sep 1;6:897. doi: 10.3389/fmicb.2015.00897 (PMC4555085; doi:10.3389/fmicb.2015.00897)

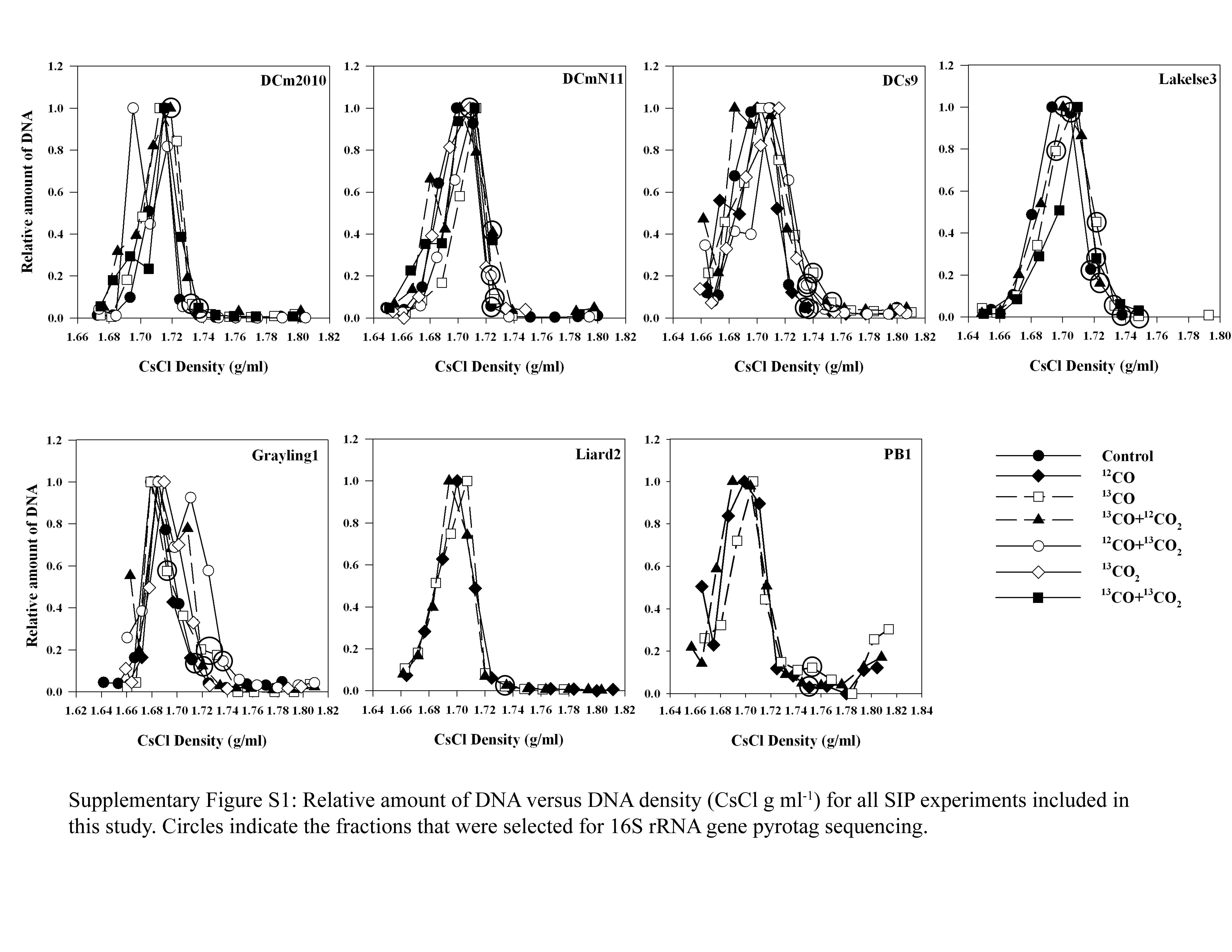

Supplement: Supplementary file 4 [file Image1.TIF]
